# Whole genome structural analysis of Caribbean hair sheep reveals quantitative link to West African ancestry

**DOI:** 10.1371/journal.pone.0179021

**Published:** 2017-06-29

**Authors:** Gordon L. Spangler, Benjamin D. Rosen, Moses Babatunde Ilori, Olivier Hanotte, Eui-Soo Kim, Tad S. Sonstegard, Joan M. Burke, James L. M. Morgan, David R. Notter, Curtis P. Van Tassell

**Affiliations:** 1 Animal Genomics and Improvement Laboratory, Agricultural Research Service, United States Department of Agriculture, Beltsville, Maryland, United States of America; 2 School of Life Sciences, University of Nottingham, Nottingham, United Kingdom; 3 Acceligen of Recombinetics Incorporated, Saint Paul, Minnesota, United States of America; 4 Dale Bumpers Small Farms Research Station, Agricultural Research Service, United States Department of Agriculture, Booneville, Arkansas, United States of America; 5 Katahdin Hair Sheep International, Fayetteville, Arkansas, United States of America; 6 Department of Animal and Poultry Sciences, Virginia Tech, Blacksburg, Virginia, United States of America; China Agricultural University, CHINA

## Abstract

Hair sheep of Caribbean origin have become an important part of the U.S. sheep industry. Their lack of wool eliminates a number of health concerns and drastically reduces the cost of production. More importantly, Caribbean hair sheep demonstrate robust production performance even in the presence of drug-resistant gastrointestinal nematodes, a rising concern to the industry. Despite the growing importance of hair sheep in the Americas their genetic origins have remained speculative. Prior to this report no genetic studies were able to identify a unique geographical origin of hair sheep in the New World. Our study clarifies the African and European ancestry of Caribbean hair sheep. Whole-genome structural analysis was conducted on four established breeds of hair sheep from the Caribbean region. Using breeds representing Africa and Europe we establish an objective measure indicating Caribbean hair sheep are derived from Iberian and West African origins. Caribbean hair sheep result from West African introgression into established ecotypes of Iberian descent. Genotypes from 47,750 autosomal single nucleotide polymorphism markers scored in 290 animals were used to characterize the population structure of the St. Croix, Barbados Blackbelly, Morada Nova, and Santa Ines. Principal components, admixture, and phylogenetic analyses results correlate with historical patterns of colonization and trade. These patterns support co-migration of these sheep with humans.

## Introduction

The U.S. sheep industry has been in steady decline since the end of WWII. The national flock peaked at about 56 million head in 1942 and dropped to 7 million by 2000[1]. However, religious, ethnic, and organic markets for lamb and sheep products have grown substantially in recent years[2]. Many consumers are attracted by the low-input, pasture-based production system of sheep. New producers are leveraging low start-up expenses and modest time commitment to maximize profitability of small production systems.

Hair sheep of Caribbean origin are ideal breeds for organic producers, especially those in sub-tropical regions. Their lack of wool eliminates the cost of shearing and obviates the need for practices such as mulesing or docking to prevent health risks particular to fleeced sheep. Heat tolerance facilitated by the characteristics of a hair coat allows these breeds to thrive in tropical, arid, and semi-arid regions[3]. High temperatures and humidity areenvironmental conditions ideal for internal parasites such as *Haemonchus contortus*. Caribbean hair sheep demonstrate robust performance even in the presence of drug-resistant gastrointestinal nematodes (GIN)[4,5,6,7]. Taken together, these factors result in reduced input and maintenance costs, decreasedlabor demands, and an overall greater return on investment than conventional wool breeds.

Despite the growing importance of hair sheep in the Americas their origins remain speculative. Prior to this report no genetic studies were able to identify a unique geographical origin of hair sheep in the New World. European, African, and Caribbean hair sheep share a common mitochondrial lineage[3], but different Y-chromosomal, and autosomal backgrounds[8]. This is logical considering the history of migration in the Old World and the colonization of the New.

During the late colonial period, sheep were transported around the globe as part of the settlement by European powers[9]. They took Merino from Spain and several British breeds to South America, South Africa, Australia and New Zealand[10]. It is often erroneously assumed these sheep mixed with indigenous breeds upon arrival to the Caribbean. However, there are no domestic sheep indigenous to the Americas.

Further confusion surrounds the African component from which these breeds originate. A typical example would be the Dorper, which is a composite breed of Dorset Horn and Blackhead Persian (DorPer)[11]. The Blackhead Persian is a fat-tailed breed and very different from the thin-tailed hair sheep of West Africa. Although classified as a hair breed, the Dorper has been shown to differ in performance from strictly Caribbean hair sheep such as the St. Croix[12], Morada Nova[13], or St. Ines[14, 15]. Another important consideration is the introgression of North African traits into the Iberian breeds developed during the Moorish occupation of Spain[16].

Recently, the greater affordability of high-throughput sequencing and high-density genotyping methodologies has facilitated impressive research into the historic development of domesticated sheep. The first draft of the sheep genome led to the development of a highly informative panel of single nucleotide polymorphisms (SNP)[17]. The International Sheep Genome Consortium (ISGC) used Hapmap data constructed from 50 thousand SNP to identify regions that have undergone positive selection across a global panel of breeds[18].

Researchers have taken the logical next step and applied these tools to elucidate the genetic structure of breeds specific to local geographic, economic, and environmental interests[19,20,21]. Dense genotyping also provides a means of mapping desired traits to specific regions of the genome designated quantitative trait loci (QTL)[22]. This identifies not only phenotypes controlled by single genes, but complex traits produced by the interaction of many genes[1, 4, 23,24].

Careful analysis of genetic structure and population stratification is an important step in understanding the genetic basis of complex traits[25]. The search for QTL depends on the identification of loci displaying outlier behavior and in itself is not proof that selection has taken place unless factors such as human-mediated selection and transportation, climate adaptation, admixture, gene flow, and introgression between breeds are taken into account[26].

With this in mind, our study attempts to clear away the ambiguity of African and European Ancestry in Caribbean hair sheep. In this study we perform whole-genome structural analysis using representative breeds from Africa and Europe to establish a quantitative link between Caribbean hair sheep and their specific origins. This is a preliminary step towards discovery of QTL linked to traits such as heat resistance, forage conversion, and parasite resistance.

## Methods

### Sample collection

Djallonke sheep are the dominant breed of West and Southwest Africa and not estimated to be endangered. Blood samples from Nigerian sheep were collected commensally during routine veterinary treatments, and all owners agreed to the analysis; no further specific permissions were required from the University of Nottingham Animal Care and Use Committee (IACUC) for this study. Ten Djallonké blood samples were taken via jugular venipuncture into Vacutainer tubes (Becton Dickinson, Franklin Lakes, NJ) containing EDTA from a privately owned flock in southwestern Nigeria with the owner’s permission. Genomic DNA was isolated from blood samples using standard procedures and stored at -20°C. Animals were genotyped using the OvineSNP50 BeadChip [Illumina, Inc., San Diego, CA]. Genotyping was performed by GeneSeek, Inc. (Geneseek/Neogen, Lincoln, NE). Raw data were analyzed using the GenomeStudio V2011.1 and Genotyping Module v.1.9.4. All call rates were greater than 99%. The genotype file was then exported to Golden Helix SNP & Variation Suite v8.x (Golden Helix, Inc., Bozeman, MT, http://www.goldenhelix.com) for further filtering and analysis.

Illumina 50K ovine genotypes of St. Croix sheep were obtained from the National Animal Germplasm Program, National Center for Genetic Resources Preservation, ARS, USDA, Ft. Collins, CO. Sample collection and DNA purification were as described by Blackburn et al[27].

### Breed selection

Using the method of Price et al[28], we first assessed the ordination of the Djallonké genome in relation to the structural variation of a global panel of 74 breeds provided by ISGC[18] (http://www.sheephapmap.org). This included three Caribbean hair sheep breeds: Barbados Blackbelly, Morada Nova, and Santa Inés and four wool breeds from the America’s: Gulf Coast Native, Brazilian Creole, Navajo Churra, and St. Elizabeth. To this we added ten St Croix genotypes obtained from the National Animal Germplasm Program (http://nrrc.ars.usda.gov/A-GRIN/main_webpage).

We then conducted a model-based Bayesian analysis[29], in conjunction with a cursory examination using SNPhyllo[30]and Treemix[31] on the complete set of 76 breeds of sheep to identify only those that may possibly have contributed to the pedigree of Caribbean hair sheep. We normalized this set to 10 individuals each to coincide with the number of Djallonké and St. Croix sheep to which our collection was limited. We finally selected only those breeds that could reasonably have contributed to the development of hair sheep through colonization and subsequent migration throughout the Caribbean (see Table 1).

**Table 1:** List of breeds and summary statistics.

Breed	Location	P_n_	H_o_	H_e_	f	A_r_	*HS_WAD_
**African Group**							
Namaqua Afrikaaner	South Africa	0.688	0.288	0.254	0.269	1.688	14.5
Ronderib Afrikaaner	South Africa	0.811	0.322	0.296	0.187	1.880	14.0
**Djallonké**	Nigeria	0.769	0.286	0.277	0.277	1.769	-
African Dorper	South Africa	0.884	0.340	0.333	0.137	1.884	11.7
Egyptian Barki	Egypt	0.948	0.369	0.371	0.066	1.948	13.2
Red Maasai	Kenya	0.884	0.326	0.325	0.173	1.884	15.6
Ethiopian Menz	Ethiopia	0.871	0.326	0.326	0.176	1.871	15.2
**Iberian Group**							
Australian Merino	Australia	0.952	0.368	0.376	0.067	1.952	11.1
Bergamasca	Italy	0.940	0.364	0.369	0.078	1.939	10.2
Castellana	Spain	0.951	0.381	0.377	0.082	1.951	10.8
Ojalada	Spain	0.953	0.378	0.377	0.045	1.953	11.2
Rasa Aragonesa	Spain	0.965	0.385	0.386	0.022	1.965	11.0
Spanish Churra	Spain	0.938	0.364	0.366	0.076	1.938	11.2
Spanish Merino	Spain	0.951	0.376	0.374	0.046	1.951	10.7
**British Group**							
Dorset Horn	UK	0.811	0.325	0.286	0.182	1.811	7.4
Dorset Poll	Australia	0.893	0.347	0.342	0.118	1.893	7.9
Soay	UK	0.745	0.269	0.268	0.317	1.745	10.0
Australian Suffolk	Ausralia	0.941	0.376	0.371	0.049	1.941	8.7
Irish Suffolk	UK	0.864	0.315	0.328	0.199	1.864	9.4
Wiltshire Horn	UK	0.752	0.272	0.263	0.309	1.752	8.0
**New World**							
Brazilian Creole	Brazil	0.946	0.341	0.371	0.136	1.946	10.8
Navajo Churro	SW USA	0.935	0.372	0.365	0.260	1.935	10.3
Gulf Coast Native	Alabama	0.951	0.373	0.373	0.055	1.951	10.7
St Elizabeth	Jamaica	0.946	0.374	0.372	0.050	1.946	13.0
**Caribbean Hair**							
Barbados Blackbelly	Barbados	0.880	0.331	0.332	0.159	1.880	23.8
Morada Nova	Brazil	0.860	0.311	0.319	0.208	1.860	21.1
Santa Inés	Brazil	0.919	0.352	0.353	0.107	1.919	23.8
St Croix	Virgin Islands	0.832	0.285	0.307	0.274	1.831	16.4
**Asia**							
Norduz	SW Asia	0.891	0.356	0.339	0.102	1.891	12.0

Intrapopulation summary statistics for 38, 337 SNP loci from 29 breeds used in this study: coefficient of inbreeding (f), fraction of polymorphic loci (P_n_), observed heterozgosity (H_O_), expected heterozygosity (H_e_), allelic richness (A_r_), and mean IBD shared between Djallonké and sample breed in Mb.

In addition to breeds representing the respective colonial influences in the region, we included several breeds as models for drift and admixture (see Table 2). The Navajo Churro is a direct descendant of the Spanish Churra brought to the Americas during the period of colonization. The Australian Merino and Australian Suffolk correspond to their British counterparts arriving in Australia sometime during the 19^th^ century[32].

**Table 2:** Control models used in this study.

**Drift**		
Spanish Churra	→	Navajo Churro
Spanish Merino	→	Australian Merino
Irish Suffolk	→	Australian Suffolk
**Admixture**		
Dorset Horn + *Corriedale/Ryland	**=**	Australian Dorset Poll
Dorset Horn +^†^Blackhead Persian	**=**	Dorper
Morada Nova+Bergamesca	**=**	Santa Inés

*The Corriedale/Ryland is represented by the Australian Suffolk.

^†^Blackhead Persian is represented by the Red Maasai/Ethiopian Menz.

The Australian Suffolk is also used to demonstrate admixture. In the U.S. the polled Dorset was the result of a mutation, but in Australia it was developed from introgression of the poll gene into the Dorset Horn from two “Down” breeds; the Morrisdale and Ryeland[33]. We use the Australian Suffolk to represent those breeds in this study. The Dorper is a composite breed developed in South Africa by crossing the Blackhead Persian from Somalia with the Dorset Horn. It is used here to demonstrate a known East African/European cross, and assist in distinguishing breeds of West African/European ancestry. We use the Ethiopian Menz and Red Maasai to represent the Blackhead Persian, which was unavailable at the time of this study. The St. Inés is a production hair sheep in Brazil developed by crossing the Morada Nova and Bergamesca[34]. The Bergamesca is a relative of the Merino imported to Brazil from Italy in the 1930s[35].

Finally, the Norduz is used in this analysis to root the phylogeny of the African breeds to the assumed Mesopotamian center of sheep domestication[36]. The Egyptian Barki is used to connect Mediterranean influence with North Africa and Iberia.

### Quality measures, summary statistics, and structural analysis

After merging all data sets we were left with 38,337 autosomal SNP from 290 individuals representing 29 breeds. Relevant diversity statistics of the coefficient of inbreeding (f) and percentage polymorphic loci (P_n_), Heterozygosity Expected (H_e_) and Observed (H_o_), and Allelic Richness (A_r_) were calculated using the PLINK version 1.9 (Purcell & Chang https://www.cog-genomics.org/plink2)[37].

Haploype sharing (HS_WAD_)was calculated using the mean size of the consensus haplotypes estimated between all pairs of individuals and between Djallonké and other breeds[38]. The haplotype phase was inferred using Beagle[38]and the summary statistics of the haplotype sharing was computed using Perl.

Removal of SNP in high linkage disequilibrium (LD) was used to counter the effect of ascertainment bias and generate meaningful comparisons among our breed populations[39]. We applied a stringent threshold of 0.05 for initial PCA analysis resulting in 5,790 sites. Generation of Eigen values from allele frequencies was performed by Golden Helix software using the Additive Model (dd)→ (Dd)→ (DD). A second analysis to remove outliers and increase resolution was performed by recomputing principal components 5 times removing outliers more than 3 standard deviations from 5 components. Admixture, phylogeny analysis, and phylogeny checks using f-statisticswere again performed on the final dataset as described above.

## Results

### Summary statistics

All statistics for the ISGC breeds were in accordance with those reported. P_n_ for Djallonké was 0.74 (see Table 1). The f-statistic for Djallonké (0.24) and St. Croix (0.29) were slightly higher than other breeds. This may be due to the single flock sampling of individuals used in this study. Caribbean hair sheep show the highest degree of haplotype sharing with the Djallonké (HS_WAD_) of any other group studied including the wool breedsfrom the same geographical region (p<0.03) (see New World group Table 1).

### Principal components analysis

We first placed the West African Djallonké, St. Croix, Navajo Churro, and Bergamesca into the complete set of genotypes provided byISGC[18]. The Bergamesca and Navajo Churro clustered indistinguishably within the group designated “Iberian/Italian/Merino” by ISGC. The St. Croix clustered with “Brazilian/Caribbean” group, and Djallonké with the “African” group. Reducing the number of individuals in each breed to 10 concentrated the clusters and did not significantly change the spatial relationship of the groups. However, this was not significant enough to resolve the relationship of the breeds of interest.

**Fig 1:**
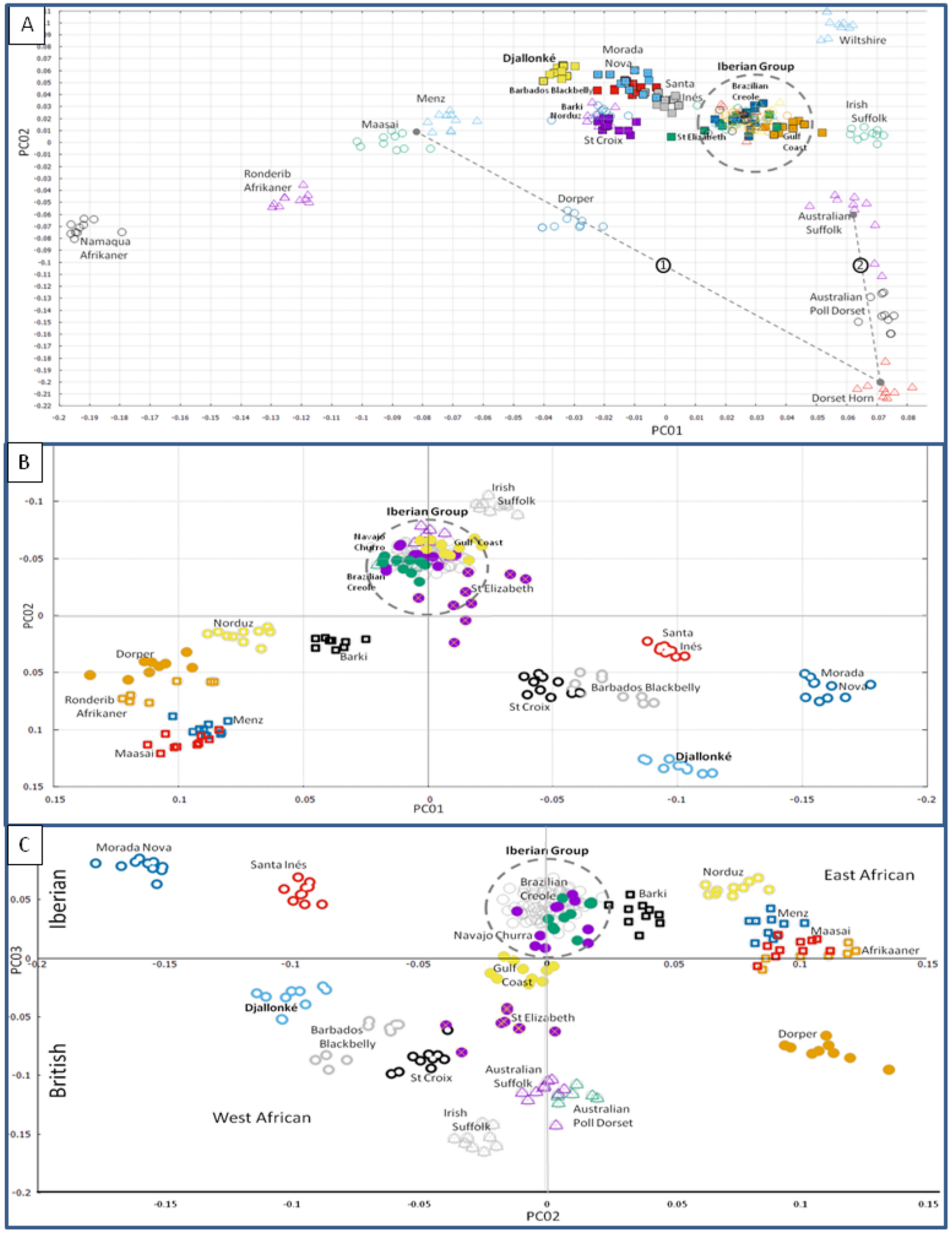
Principal components analysis. **A.** *Multi-Dimensional Scaling (MDS) plot of the pair-wise Identity by State distances among the 27 breeds used in this study*. The Iberian Group is indicated by a dashed circle and contains the New World Breeds: Navajo Churro, Brazilian Creole, Gulf Coast Native, and St Elizabeth. The Spanish and Australian Merino are indistinguishable in this group, as well as the Spanish Churra, Ojalada, Rosa Aragonesa, Castellana, and the Italian Bergamesca. Dashed lines indicate breeds of known admixture: (1)- The Dorper is a composite breed of the Dorset Horn and Blackhead Somali (Menz/Maasai used as substitutes). (2)- The Australian Poll Dorset was developed by inserting the poll gene into the Dorset Horn by crossing with Corriedale and Ryeland, a breed similar to the Australian Suffolk. **B.** *MDS plot of Components 01 vs 02 after outlier removal*. Component 1 clearly separates the West African Djallonké and the four Caribbean Hair sheep from the East African group: Menz, Maasai, and Afrikaaner. PC02 separates the Hair sheep from the European breeds leaving the Caribbean Hair sheep in a quadrant alone with the West African Djallonké. The Dorper is approximately 70% East African admixture and clusters with this group. All of the European breeds cluster in or near the Iberian Group along with the New World wool breeds. **C.** *MDS plot of Components 02 vs 03 after outlier removal*. As in the above plot PC02 divides East and West African breeds. PC03 further separates the British from the Iberian.

We therefore reduced the number of breeds to those which could have reasonably contributed to Caribbean ancestry and reran the analysis (Fig 1A). The cline demonstrated by the African breeds in PC01 and PC02 concurs with known migration of fat-tailed, course-wooled sheep into the continent from the origin of domestication[40], and the spatial orientation of these breeds roughly matches the geographic location of the sampling locations[41]. The Djallonké skews slightly higher in PC02 aligning it in a position of admixture with the Caribbean breeds and the Iberian. The position of the Dorper directly between the Menz/Maasai and Dorset Horn suggests this is a valid assumption. Further reinforcement of this assumption can be seen in the gradient demonstrated among Dorset Horn, Australian Poll Dorset, and Australian Suffolk. A shift between the Irish and Australian Suffolk is most likely due to admixture rather than drift, as the Spanish and Australian Merino are indistinguishable within the Iberian group. This is also the case with the Spanish Churra and Navajo Churro.

To further increase resolution we reran the analysis this time eliminating outliers (Fig 1B and 1C). This eliminated the Namaqua Afrikaaner, Wiltshire, and Dorset Horn from the data set. PC01 versus PC02 clustered all the European breeds except for the Irish Suffolk within the Iberian group. The New World fleeced breeds also clustered within this group. PC01 distinctly separated all of the remaining East African breeds from the West African placing it squarely within the quadrant containing only the Caribbean hair breeds. The Dorper which is a composite of approximately 70% Blackhead Somali (Maasai/Menz) clustered with the East African breeds.

PC02 versus PC03 (Fig 1C) maintained the separation of East and West Africa but PC03 separated the European group into British and Iberian breeds. The Morada Nova and Santa Inés clustered on the Spanish side, while the St. Croix and Barbados Blackbelly clustered to the British side. The African Dorper, Gulf Coast Native, and St. Elizabeth were also clustered to the British side while the Navajo-Churro and Brazilian Creole remained clearly within the Iberian group.

### Admixture

**Fig 2:**
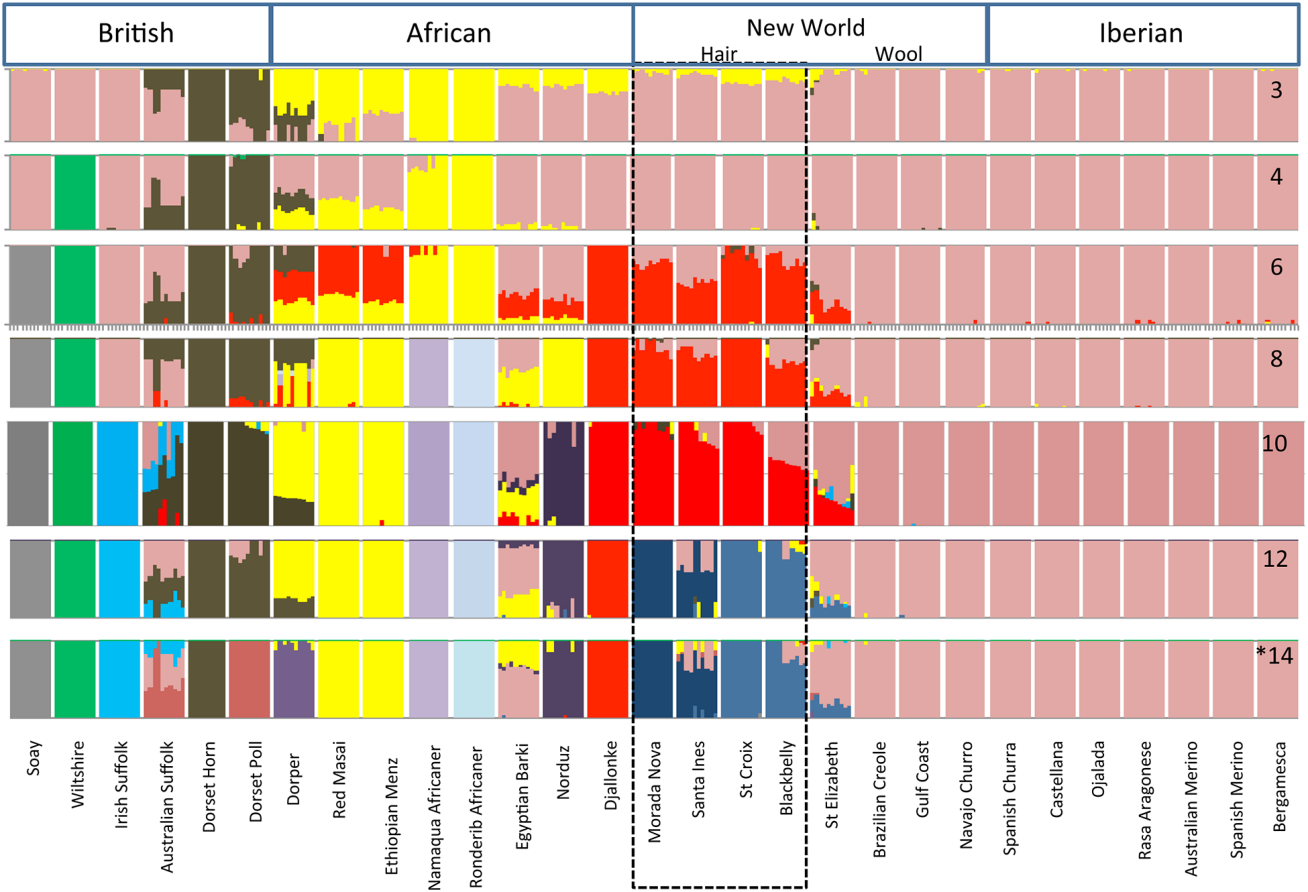
Admixture analysis of 29 breeds used in this study. Breeds are separated into blocks. Lines within each block represent individuals within the breed. Graphs for K = 3–14 show the admixture ratio of each theoretical population [K] within the individuals in a block. Groups are indicated at the top. Caribbean hair sheep are inscribed by dashed lines. Norduz is included with the African group for simplicity.

The genetic structure of Caribbean hair sheep breeds was investigated by model-based Bayesian analysis using the software package fastStructure[29]. Results from K = 3 to 14 are shown in Fig 2. LD restrictions were relaxed to threshold level of 0.8 leaving 17,138 SNP. We tested theoretical population values from 2 to 24. After which a maximum likelihood value of optimal differentiation was calculated as K = 14. The pattern of differentiation generally follows that of Kijas et al[18] for breeds obtained from ISGC. However, the addition of the Djallonké and St. Croix clearly indicates a significant West African component in the Caribbean breeds.

K = 3 to 6 shows differentiation of the ancient breeds Dorset, Afrikaaner, Wiltshire, and Soay. However, we see a marked separation of the African groups with the addition of the Djallonké to this panel beginning at K = 6. Namaqua, Ronderib, Maasai, Menz, and Dorper segregate to the East African side, and all the Caribbean hair breeds cluster with the West African Djallonké. The fleece breed from St. Elizabeth (Jamaica) also shows some admixture with the West African samples. The American wool breeds Navajo Churro, Brazilian Creole, and Gulf Coast Native remain with the Iberian group.

At K = 10 the genetic components of the Dorper agree with the documented make-up of this composite breed of approximately 70% East African and 30% Dorset Horn. At K = 12 we see indications of the mixtures used between the Australian Suffolk and Dorset Horn to create the Australian Poll Dorset. This mixture of shared genetic components demonstrates the contribution of the Australian Suffolk relative to the Irish Suffolk rather than geographic separation of the two. For this reason we also included the Spanish and Australian Merino and the Spanish and Navajo Churro, which show no cross mixture of components.

At K = 12 we see a divergence of the Caribbean Hair breeds St. Croix and Barbados Blackbelly from the Morada Nova and Santa Inés. At K = 14 we see the full elucidation of the Santa Inés, which was developed from the Morada Nova using Bergamesca and Blackhead Somali[34].

### Treemix

**Fig 3:**
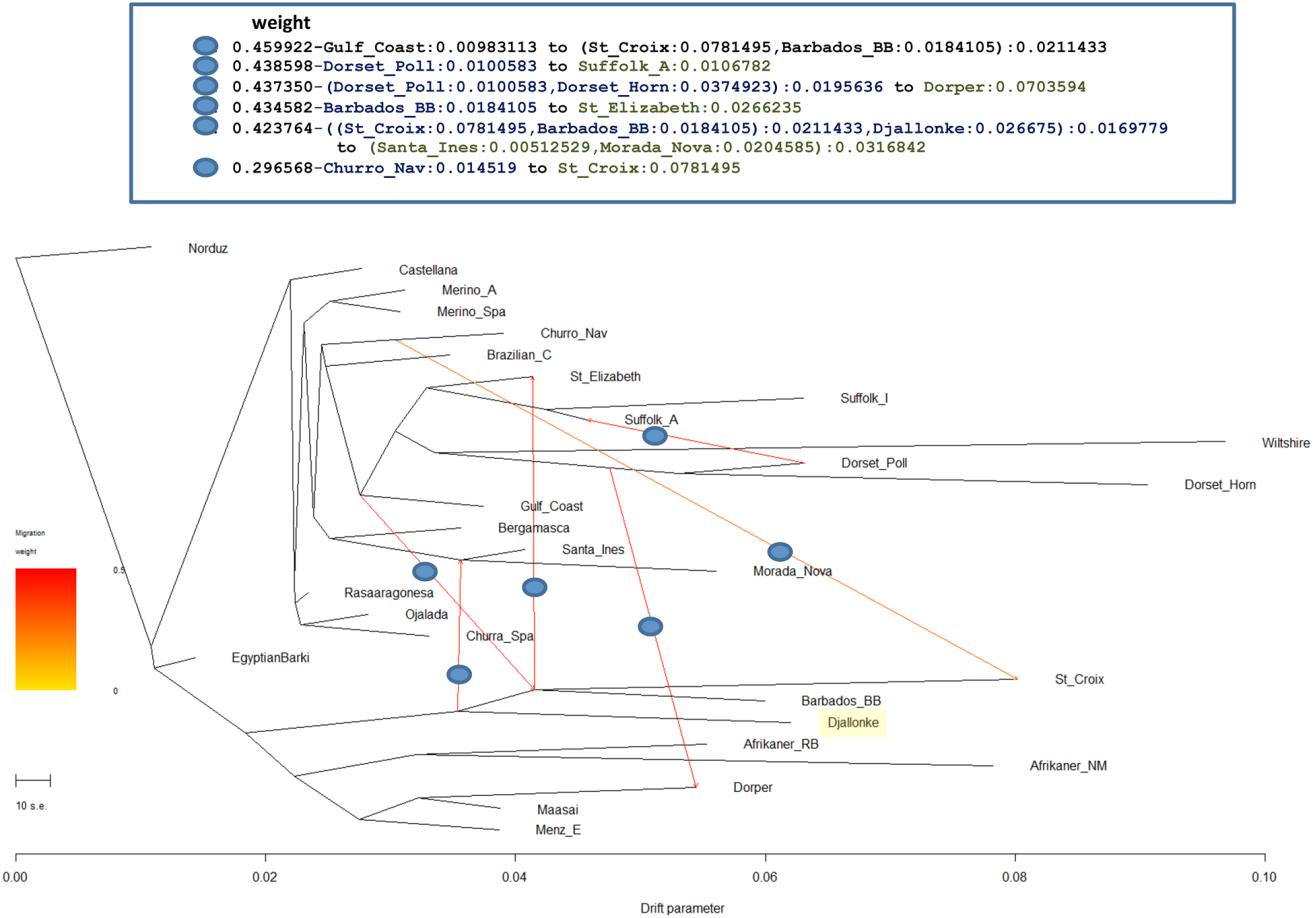
Tree topology with six migration events inferred by Treemix. Drift parameter is shown at the bottom and weights of inferred gene flow shown at the top in modified Newick format.

TreeMix uses allele frequencies to infer relationships amongpopulations through the construction of a graph. The graph allows modeling of population splits as in traditional bifurcating tree methods but has the added benefit of indicating gene flow among groups (Fig 3)[31]. We used f-statistics to validate migration edges in the tree (Supplemental Table 1). The f3 and f4 statistics test for the validity of connections in three or four population trees respectively. The f3 statistic showed significance for admixture in the Australian Suffolk, however all migrations identified showed significance with f4 statistics.

We rooted our phylogeny on Norduz, which is the breed sampled closest to the center of domestication geographically. The nearest neighbor to the Norduz is the Barki of North Africa reflecting the accepted migration pattern of fat-tailed sheep into the continent[42]. After this the tree bifurcates into African and Iberian breeds.

The African branch splits East and West. The Ronderib and Namaqua Afrikaner are a more ancient branch, and Namaqua demonstrates considerable drift. The Dorper is in a clade with Menz and Maasai and receives a highly weighted migration event from the Dorset family. This correlates to the known composition of Blackhead Somali and Dorset to develop this breed [11]. The West African Djallonké shares a branch with the Barbados Blackbelly and St. Croix proving its significant influence on these hair sheep breeds and a strongly weighted migration event into the Morada Nova/Santa Inés branch.

Castellan leads to the Rasa Aragonesa, Ojalada, and the Spanish Churra. The Spanish Merino and Australian Merino share the same branch, which supports the idea that the PCA and fastStructure analyses are biased relative to Suffolk. The Navajo Churro, Brazilian Creole, and Gulf Coast Native are in the Iberian branch and no indication of African gene flow into these breeds is seen. Following in this clade are the Bergamesca, Morada Nova, and Santa Inés, which agrees with Paiva et al[34].

The British group ties into the Iberian group through St. Elizabeth and the Gulf Coast Native. Sheep of the British Isles developed through a northern migration from the center of domestication and therefore display less admixture with those of Africa or Iberia[36]. Jamaica (St. Elizabeth) and the Gulf Coast were colonized by the British and this would logically be where the two influences meet.

## Discussion

We have established the first quantifiable link between Caribbean hair sheep and their West African ancestors. Previous investigation using mitochondrial and microsatellite markers were unable to specify a single source for the origin of hair sheep in the region[43,44]. Most agree the major influence on Caribbean hair sheep is Iberian, but fail to account for introgression of West African traits[45, 46].

We have shown which breeds share African genetic components and to what degree. Furthermore, we have eliminated some breeds previously assumed to contain African admixture. Our whole genome structural analysis shows that Caribbean hair sheep are descendants of Spanish breeds, most likely the Churra, and West African breeds. The New World wool breeds are of Spanish descent without West African introgression.

Hair sheep are important for the economic viability they bring to the sheep industry, and especially for their remarkable resilience in the presence of multi-drug resistant parasites such as *H*. *contortus*. Many in-depth studies searching for genomic loci responsible for these traits have produced inconclusive results. We chose instead to clarify the ancestry of these breeds using structural genomic analysis taking into account the dynamic historical colonization of the region.

**Fig 4:**
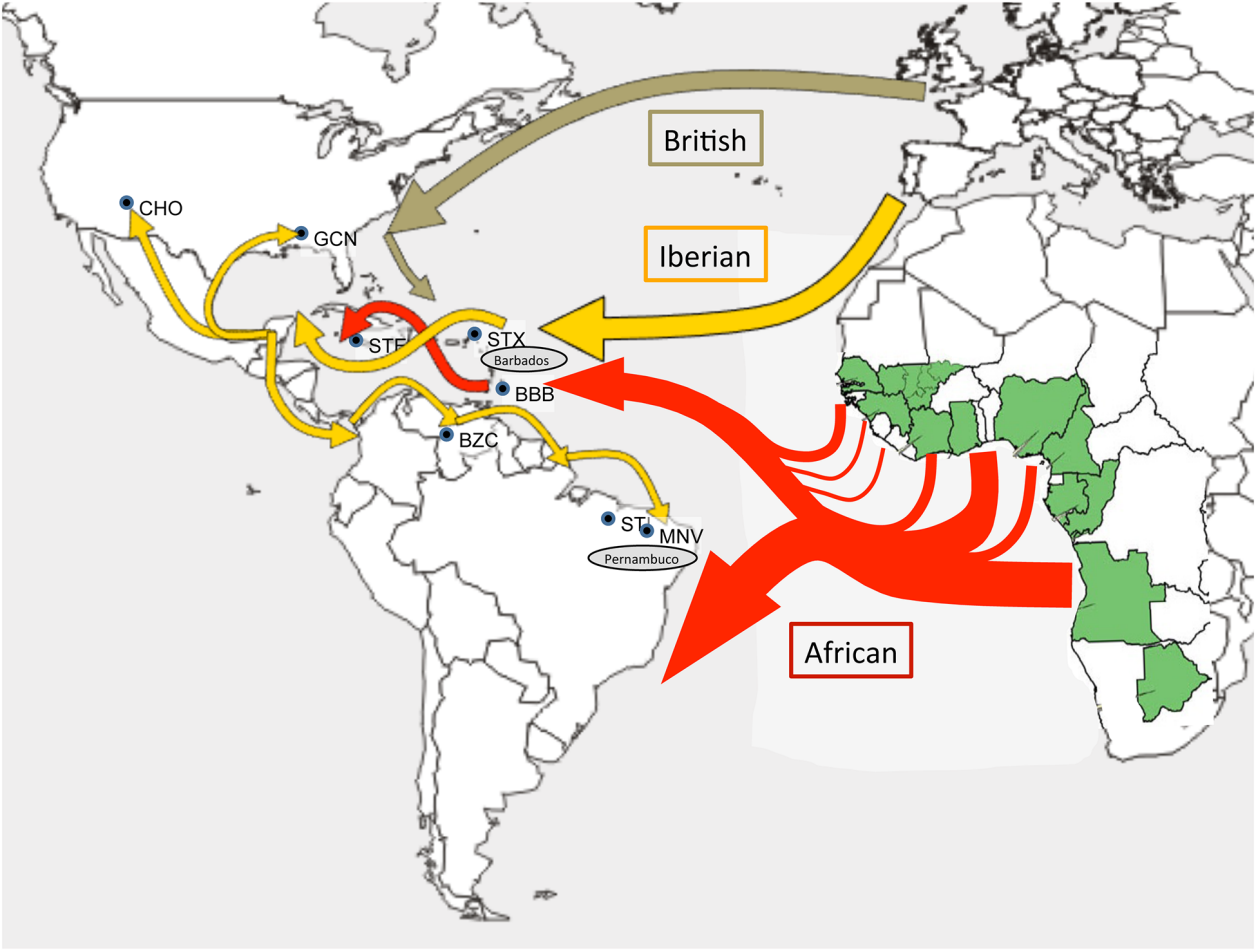
Historical and geographical corroboration of the results of whole genome structural analysis. Map indicates migration routes of British (olive) and Iberian (Orange) colonization and locations of New World and hair sheep breeds used in this study; Navajo Churro (CHO), Gulf Coast native (GCN), St. Elizabeth (STE), St. Croix (STX), Barbados Blackbelly (BBB), Brazilian Creole (BZC), Morada Nova (MNV), Santa Ines (STI). Red arrows indicate routes and relative magnitude of the Trans-Atlantic Slave Trade taken from Galloway. Gray ovals (Barbados and Parambuco) indicate regions of heavy sugar production and centers of slave importation. Green highlighted African nations indicate range and distribution of the West African Djallonké sheep (FAO).

Spanish Churra arrived in the Caribbean at the beginning of the 16^th^ century with the second voyage of Columbus[47] (Fig 4). It is a hardy, coursewooled, thin-tailed breed accustomed to long cold winters and hot dry summers. It was developed in Spain along with its close cousin the Merino during the Arab Agricultural revolution from the 8^th^ to the 13^th^ centuries[16,48]. However, due to the extreme value of its wool at the time, Merinos were banned from exportation during the early stages of colonization.

The Churra were spread ubiquitously throughout the Caribbean, co-migrating with explorers and settlers (Fig 4, Orange arrows). Livestock, including sheep, were often left to fend for themselves as a food source for subsequent expeditions. Colonization towards mainland America followed shortly after, spreading north and south from the Yucatan peninsula. Northward expansion into present day southwestern U.S. was taken up by the indigenous peoples and resulted in the Navajo Churro. Northeastern migration around the Gulf of Mexico met with the southwestern expansion of the British colonies. Southward migration from Spanish Mexico proceeded down the Pacific coast of South America with a retrograde movement into southern Brazil.

There is virtually no difference in our structural analysis between the Navajo Churro and its accepted ancestor the Spanish Churra. Its principal components oriented indistinguishably from the other breeds in the Iberian group under all conditions. It also clustered identically with the Spanish Churra in admixture analyses. Nearest neighbor trees placed it in the same clade with the Iberian sheep showing only slight drift. This is remarkable considering a difference of 500 years between the two breeds.

Our results agree with the accepted belief that the Brazilian Creole is a product of the retrograde movement along the Pacific and Northeastern coasts of South America[49,50]. The timing and pattern of its migration never brought it into contact with the introgression of West African sheep into the region. The Gulf Coast Native reflects a similar pattern; populating the North American coast of the Gulf of Mexico it would have met with the additional influence of the British. Our PCA shows it leaning in the direction of the British breeds. Although our scaled down Bayesian analysis indicated no British admixture (Fig 2), more comprehensive analyses do show anapproximate 10% British component[18]. British breeds evolved and migrated along different lines than Mediterranean sheep[36,51,52]. Our nearest-neighbor tree shows the British branch joining at the junction of the Gulf Coast Native and St. Elizabeth indicating these breeds as the closest link between the British and Iberian groups in this study.

Our genomic analysis clearly indicates a major influence of West African lineage on Caribbean hair sheep. This is strongly supported by the pattern of colonization, economic structure, and trade routes of the period. The introduction of West African sheep into Caribbean populations probably began approximately 100 years after the arrival of the Iberian sheep as the search for silver and gold gave way to sugar production. The rise of the sugar industry created a desperate demand for labor. Historical as well as genomic data shows that this demand was filled by the enslavement of peoples from the West Coast of Africa[53]. The amount of sugar produced in a region roughly correlates to the number of slaves imported to that region (Fig 4 red arrows adapted from [54]). The nature of the Triangle Trade and the reliance on the trade winds prescribed the region of Central West Africa known historically as The Slave Coast. It roughly stretches from the modern day countries of Senegal to Angola. This is the exclusive range of the West African Dwarf or Djallonké breeds of sheep (Fig 4 green areas. Map adapted from from [55]).

Main centers of importation in the Caribbean were the Northeast coast of Brazil and the Eastern Islands of the Caribbean, especially those ports connected to centers of sugar production such as Parambuco and Bahiain Northeastern Brazil, and the islands of Barbados and Jamaica in the British controlled Caribbean (Fig 4 gray ovals) [54]. It is at these junctions where the influx of West African sheep carried coincidentally along with the slave trade met and mated with the well established descendants of the Iberian Churra. The Morada Nova, Santa Inés, St. Croix, and Barbados Blackbelly are breeds named after the locations of their development. These correspond exactly to regions of highest slave importation and by correlation, West African sheep.

All of our Principal Component analyses indicate a link to the West African Djallonké. Outlier removal places the West African squarely in the quadrant exclusively occupied by the Caribbean hair sheep. Admixture analysis from K = 6 to 10 shows each to be a clear product of Iberian and West African ancestry. Although, these breeds differentiate above K = 10 it has been shown previously that theoretical population values of 10 or less are best indicators of ancestry in this type of analysis[25]. Treemix places the St. Croix and Barbados Blackbelly in the same clade as the Djallonké. Although, Morada Nova, and Santa Inés are in line with the Spanish Churra they show a strong indication of gene flow from the West African clade.

Caribbean hair sheep are a result of the introgression of West African traits into the ancestors of the Spanish Churra deposited by Iberian colonists from the time of Columbus. Further differentiation among the Hair sheep breeds may be a result of the influence of British breeds on the Northern Caribbean populations, the degree of husbandry, or simple climate-mediated adaptation. Caribbean hair sheep with West African heritage are ecotypes that combine the benefits of hair coat and disease resistance with economic viability. These locally adapted breeds represent reservoirs of adaptive fitness traits that can contribute to the future of sustainable sheep farming in the face of economic change, global warming, and the increasing pressure of drug-resistant parasites.

## Supporting information

S1 Table Table of f3 and f4 tests for validation of Treemix gene migrations. (XLSX)
